# The roles of quantitative doppler echocardiography in optimizing guideline-directed medical therapy in Post-STEMI patients

**DOI:** 10.1007/s10554-026-03722-9

**Published:** 2026-04-27

**Authors:** Juan Lei, Tou Kun Chong, Jingwei Gao, Jian Chen, Kan Liu

**Affiliations:** 1 Division of Cardiology, Sun Yat-sen Memorial Hospital of Sun Yat-sen University, Guangzhou, People’s Republic of China; 2 Department of Cardiology, Kiang Wu Hospital, Macao, People’s Republic of China; 3 Department of Cardiovascular Medicine, The Fifth Affiliated Hospital, Sun Yat-sen University, 52 East Meihua Rd, Zhuhai, 519000 People’s Republic of China; 4 Division of Cardiology, Heart and Vascular Center, Washington University in St Louis, BarnesJewish Hospital, 4523 Clayton Ave, St. Louis, MO 63110 USA

**Keywords:** ST-segment elevation myocardial infarction, Evidence-based pharmacotherapy, Hemodynamic instability, Point-of-care ultrasound, Quantitative Doppler assessment, Preload titration

## Abstract

Patients with ST-segment elevation myocardial infarction (STEMI), who are at the highest risk, benefit the most from early guideline-directed medical therapy (GDMT). In real-world practice, timely application and titration of these medications are often compromised during the immediate post-STEMI period due to the perception of hemodynamic instability. An extended medication gap may last from the early post-STEMI period until outpatient follow-up weeks later, depriving prognostic benefit from key pharmacotherapy in the critical window of early post-infarct course. We discuss the practical hurdles in the post-STEMI period to commence GDMT, and the tactics to optimize risk management in high-risk patients to realize the benefits of GDMT. We highlight the challenges of real-time cardiac preload evaluation after coronary revascularization, and the emerging roles of quantitative Doppler echocardiography a point-of-care non-invasive imaging approaches in guiding preload titration to support early pharmacotherapy. We propose stepwise algorithms to integrate instantaneous quantitative Doppler assessment into routine post-STEMI management workflow, and outline our perspective for future trial design and clinical practice.

**Supplementary Information:**

The online version contains supplementary material available at https://doi.org/10.1007/s10554-026-03722-9.

## Introduction

In the United States, the annual incidence of acute coronary syndrome (ACS) is estimated to be 605,000 for new cases and 200,000 for recurrent cases. More than 35% of patients presenting with ACS have an ST-segment elevation myocardial infarction (STEMI) [1–3]. Although prompt coronary revascularization has significantly reduced mechanical complications, more than 100,000 STEMI patients continue to die after myocardial infarction each year [1]. Post-STEMI pharmacotherapy remains important in improving short- and long-term outcomes. Early prospective trials have demonstrated that immediate utilization of angiotensin converting enzyme inhibitors (ACEIs) reduces post-STEMI mortality [4–7]. Beta-blockade (BBs), mineralocorticoid receptor antagonists (MRAs) and angiotensin receptor-neprilysin inhibitor (ARNI) has also been shown to favorably impact long-term prognosis [8, 9]. Early initiation of Sodium-glucose cotransporter-2 inhibitors (SGLT2i) in acute myocardial infarction was associated with reduced risk of heart failure hospitalization [10]. Recently, evidence-based pharmacotherapy was proven to improve prognosis in post-STEMI patients even without traditional risk factors, such as hypertension, dyslipidemia and diabetes [11].

Nonetheless, in real-world practice, use of these medications in high-risk patients are often limited in the immediate post-STEMI period due to concern for worsening hemodynamic instability. As a result, a prolonged delay in medication initiation may persist from the acute post-STEMI phase through to outpatient follow-up, potentially missing the critical early period following infarction when timely pharmacological treatment could offer significant prognostic advantages [12, 13]. Therefore, we discuss the challenges of real-time cardiac preload evaluation in high-risk STEMI patients after coronary revascularization, and the emerging roles of point-of-care non-invasive imaging approaches in guiding cardiac preload titration and support evidence-based pharmacotherapy. We propose stepwise algorithms to integrate instantaneous quantitative Doppler assessment into the routine post-STEMI management workflow, and outline our perspective for future trial design and clinical practice.

## Post-STEMI risk stratification and pharmacotherapy

Based on the databases of large clinical trials and registries, multiple predictors have been identified to help risk assessment in the early post-STEMI period. Other than older age, lower left ventricular ejection fraction (LVEF), hypotension and tachycardia were found to predict the highest 30-day and one-year mortality rates among STEMI survivors [14–16]. From the data of community-based patient populations, lower LVEF, hypotension and tachycardia were also validated to predict worse in-hospital and long-term outcomes [17, 18]. Similar results have been shown in STEMI patients with moderate to severe coronary artery disease, which reflect more contemporary cardiology experience [19].

**Fig. 1:**
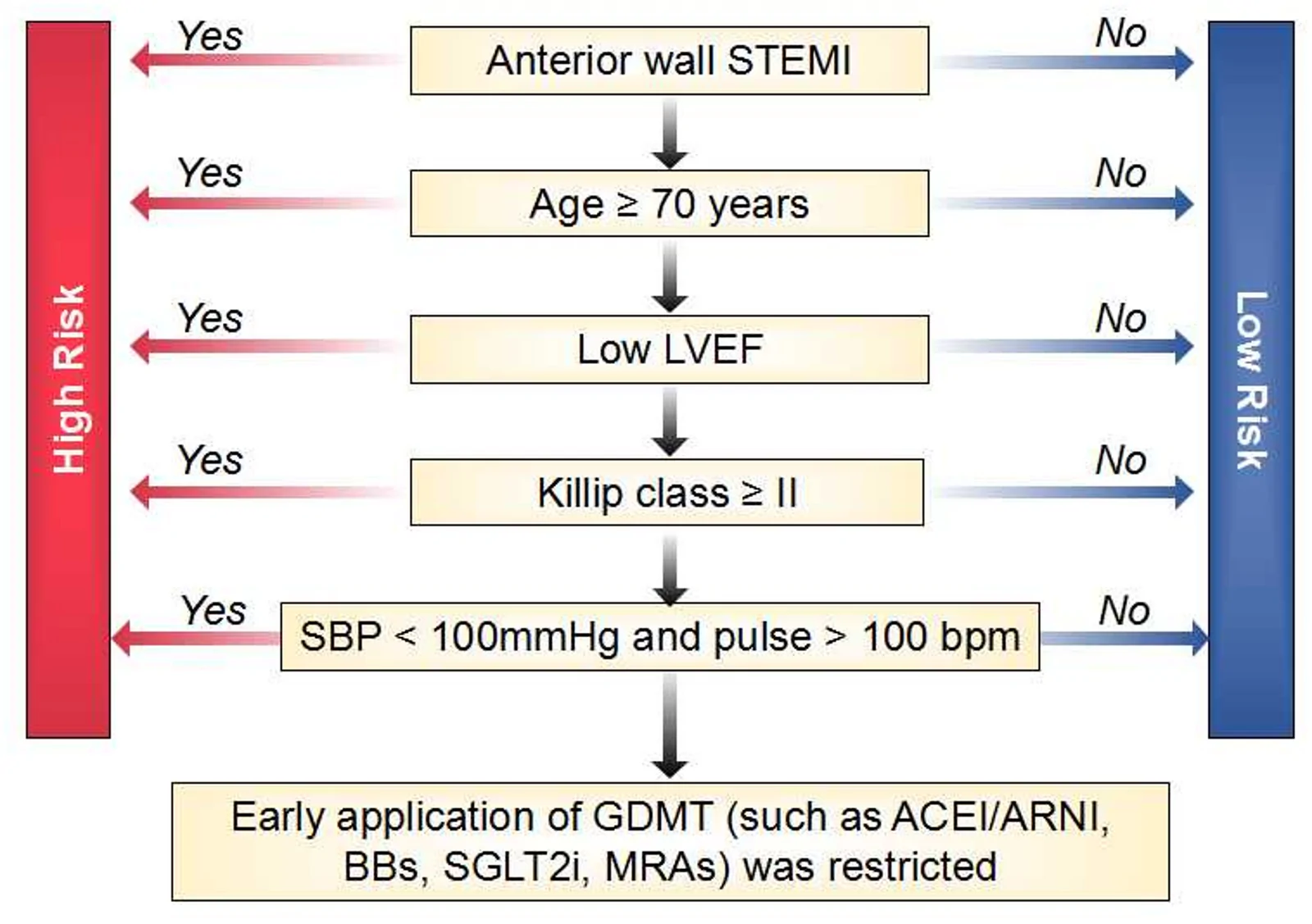
High-risk STEMI patients who were restricted from the early application of evidence-based pharmacotherapy. ACEIs: angiotensin converting enzyme inhibitors; ARNI: angiotensin receptor-neprilysin inhibitor; BBs: beta blockers; LVEF: left ventricular ejection fraction; MRAs: mineralocorticoid receptor antagonists; SBP: systolic blood pressure; SGLT2i: Sodium-glucose cotransporter-2 inhibitors; STEMI: ST-elevation myocardial infarction

Meanwhile, prospective trials have demonstrated that early utilization of GDMT in post-STEMI patients could improve short- and long-term prognosis (Fig. 1) [4–9]. Prognostic benefit was achieved with BBs in STEMI patients even before coronary revascularization and even without revascularization [12, 13]. Of note, the benefits of evidence-based pharmacotherapy principally occurred in patients with severely decreased LVEF [7, 12, 20], proving the “leitmotif” of “the sickest hearts benefit the most.” Nonetheless, in real-world practice, many high-risk patients who are expected to benefit the most from evidence-based pharmacotherapy in the early post-infarct period are paradoxically “intolerable” of them. Further, clinicians are frequently challenged with the judgment of whether hypotension represents inadequate ventricular preload versus true cardiac pump failure, as pharmacologic management would be different for each scenario.

## Faulty hemodynamic judgment during post-STEMI period

The assessment of LVEF is crucial for the application of GDMT. However, myocardial contractile function and LV filling pressure are affected by many pathophysiological and iatrogenic factors. Contrary to a common misconception, post-STEMI patients with reduced LVEF do not always have high left ventricular (LV) filling pressure [21, 22]. The current understanding of clinical acute heart failure (HF) secondary to anterior wall STEMI largely centers round LV function; nonetheless, an in-depth appreciation and classification of hemodynamic patterns can lead to a better tailoring of acute HF treatment according to hemodynamic subcategories in post-STEMI patients [23, 24].

Clinicians may rely on physical examination, plasma biomarkers, and imaging findings to evaluate volume status, achieving accurate hemodynamic assessment remains challenging in post-STEMI patients who have comorbidities such as obesity, chronic venous insufficiency, renal impairment, or underlying lung disease. One frequent source of misjudging left ventricular filling pressure is the discrepancy between right and left ventricular pressures (R-L mismatch) [21, 22]. For instance, the interpretation of jugular venous distention assumes a correlation between right and left ventricular filling pressures. However, this assumption can be invalidated in the post-STEMI setting when pre-existing pulmonary hypertension distorts the relationship, leading to inaccurate clinical assessments based on jugular vein evaluation. For similar reasons, our decision-making on volume management in post-STEMI patients can potentially be misled by co-existing RV dysfunction [25], tricuspid regurgitation [26], obstructive sleep apnea [27], atrial fibrillation [28], and dynamic left ventricular outflow obstruction [29]. These pathologies are usually more prominent in elderly patients, one of high-risk STEMI patient subgroups.

If LV volume overload is assumed to be the cause for decompensated symptoms (such as dyspnea) in post-STEMI patients with low LVEF, preload reduction easily becomes the main pharmacotherapy focus. In real-world practice, transiently elevated LV end diastolic pressure (LVEDP) obtained during cardiac catheterization is often used as a surrogate for persistent LV volume overload during the post-STEMI period [30], leading frontline clinicians to continue diuretics. However, the elevated LVEDP is usually transient after successful coronary revascularization, and high-risk patients may benefit from slowing of diuresis or volume repletion to allow institution of ACEIs/ARNI, BBs, MRAs and SGLT2i [7–10].

## Hypotension and inadequacy of evidence-based pharmacotherapy

**Fig. 2:**
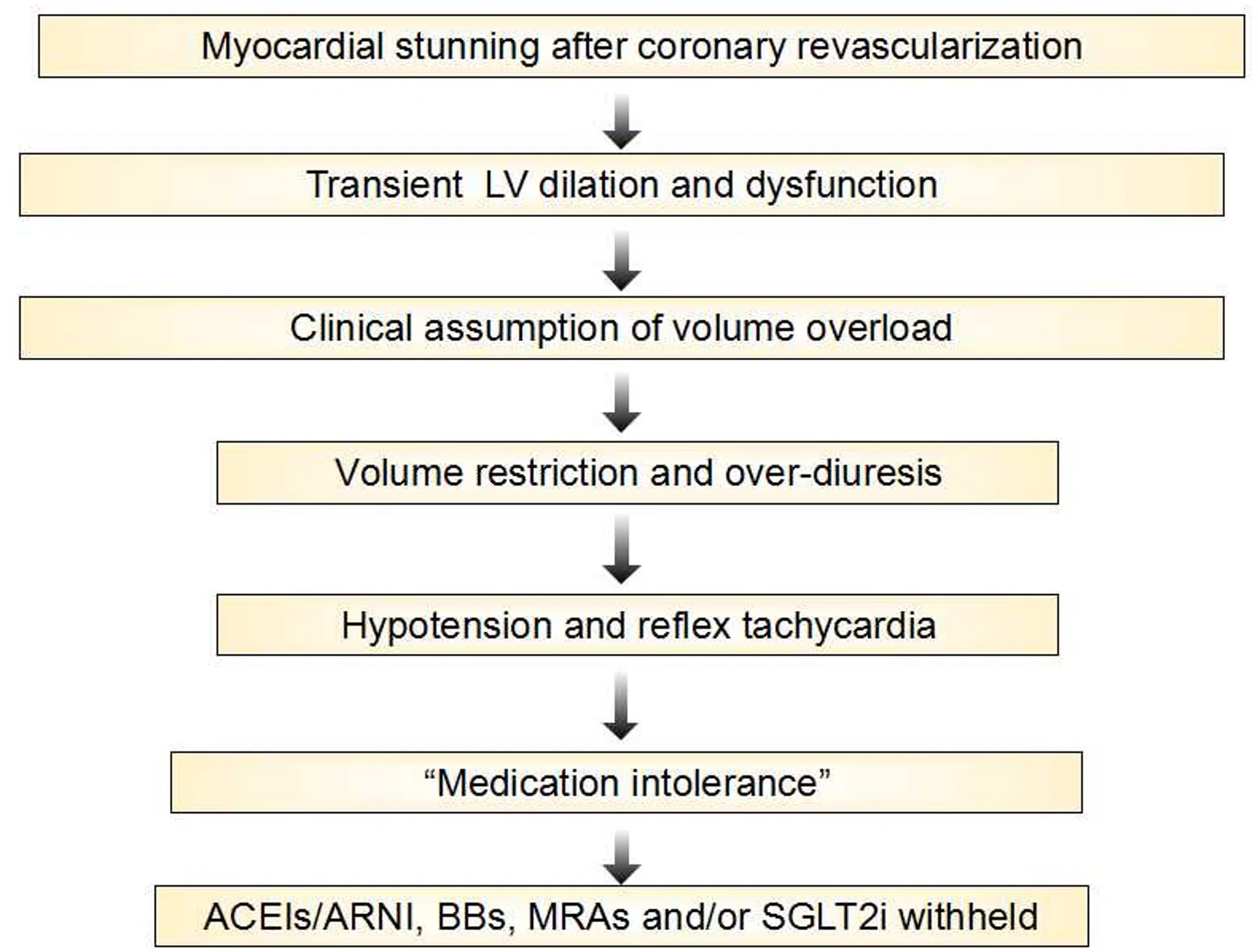
Inappropriate preload management and medication intolerance. ACEIs: angiotensin converting enzyme inhibitors; ARNI: angiotensin receptor-neprilysin inhibitor; BBs: beta blockers; LV: left ventricular; MRAs: mineralocorticoid receptor antagonists; SGLT2i: Sodium-glucose cotransporter-2 inhibitors

Other than over-diuresis, there are additional pathophysiological factors which may contribute to post-STEMI hypotension and tachycardia. The physiological stress induced during STEMI activates both the central and autonomic nervous systems, leading to increased fluid loss from the body. In addition to serving as a prognostic marker, the release of brain natriuretic peptide (BNP) during STEMI promotes diuresis, induces vasodilation, and suppresses the renin-aldosterone system, resulting in reduced mean arterial pressure and lower pulmonary capillary wedge pressure [31]. Natriuresis, when combined with diuresis, may cause post-STEMI patients to become volume sensitive, precipitating hypotension and reflex tachycardia (especially when atrial fibrillation co-exists), and pre-renal dysfunction. Conversely, even following successful coronary revascularization, the presence of apparent myocardial stunning observed on ventriculography or echocardiography may be misinterpreted as ongoing pump dysfunction and fluid overload. This perception can lead to excessive fluid restriction and aggressive diuresis, resulting in suboptimal left ventricular preload for the stunned myocardium and potentially precipitating hemodynamic compromise. Consequently, clinicians may hesitate to initiate ACEIs/ARNI, BBs, MRAs and/or SGLT2i early after STEMI, delaying guideline-recommended therapies (Fig. 2).

Given the pressure to reduce length of hospital stay, post-STEMI patients often end up being discharged prematurely without (or with inadequate dosage of) GDMT. This lack of therapy will likely last until their first outpatient follow-up visit several weeks later, therefore missing the window of early institution of GDMT, the key components of beneficial outcome therapy in high-risk STEMI patients [5–8]. Faulty real-time hemodynamic assessment and management can result in a “domino effect”, preventing maximal prognostic benefits of STEM patients from evidence-based pharmacotherapy.

## Real-time quantitative doppler assessment to support early post-STEMI pharmacotherapy

Routine bedside echocardiography, or point-of-care ultrasound (POCUS), is recommended in high-risk post-STEMI patients in updated guidelines/consensus to evaluate post-infarct ventricular and valvular function, as well as to exclude mechanical complications and ventricular thrombus [32]. Simultaneous Doppler measurements using POCUS can be synergistic with regard to workflow efficiency. Due to its noninvasive and portable features, quantitative Doppler assessment becomes a valuable bedside tool for monitoring real-time hemodynamic status and therapeutic response and guiding post-STEMI medical management [33, 34]. There is accumulating evidence that the accuracy of quantitative Doppler measurement on LV filling pressure is comparable to that obtained by pulmonary artery catheterization, particularly in patients with reduced LVEF [35, 36]. The EURO-FILLING Study, a multi-national prospective trial [37] further validated that assessing LVEDP non-invasively was reliable and clinically useful, and even superior to invasive measurements [38]. Using quantitative Doppler, a non-invasive method, instead of pulmonary artery catheterization, which is invasive, will avoid the costs and complications associated with invasive examinations. In addition to structural and functional assessment with echocardiographic images, front-line clinicians can use Doppler signals to analyze instantaneous pressure changes of all cardiac chambers, teasing out the often-nuanced interdependence of volume, pressure and contractile function, thereby better comprehending the otherwise puzzling real-time post-STEMI pathophysiology [39]. Some scholars have started to explore the application of bedside echocardiographic monitoring to guide the treatment of hospitalized patients with acute heart failure [40].

**Fig. 3:**
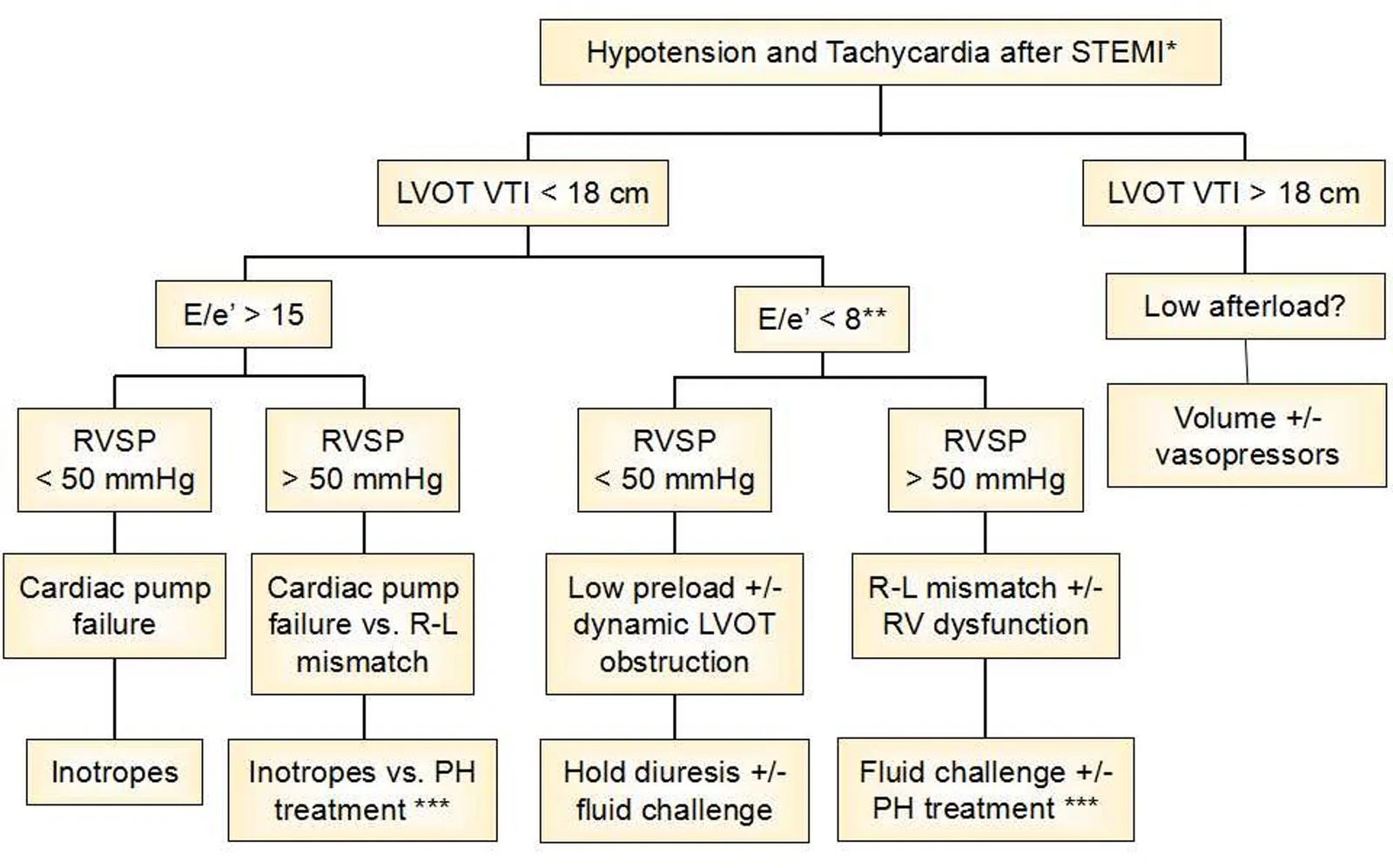
Quantitative Doppler-guided post-STEMI hemodynamic management in high-risk patients. E: early diastolic transmitral peak velocity; e’: early diastolic velocity of the myocardium at the level of the mitral annulus; LVOT: left ventricular outflow tract; PH: pulmonary hypertension; RV: right ventricular; RVSP: right ventricular systolic pressure; VTI: velocity time integral. *“Hypotension” is defined by mean blood pressure < 60 mmHg or systolic blood pressure < 90 mmHg; tachycardia is defined by heart rate higher than 100 bpm. ** An E/e’ ratio between 8 and 15 represents a “gray zone” in clinical practice. Its accuracy in predicting LVEDP remains controversial. In such cases, a comprehensive analysis that incorporates the clinical context and other parameters is required. ***PH under these conditions is commonly caused by chronic obstructive pulmonary disease, sleep disorders, and chronic thrombotic and/or embolic disease (Classes III and IV of PH). (This figure is reproduced by permission. License number: 6192010941294)

Although various quantitative Doppler techniques can aid in evaluating intracardiac and intrapulmonary pressures [33], the key hemodynamic data required to detect right-left ventricular pressure discordance (R-L mismatch) can be derived by concurrently assessing three guideline-recommended Doppler parameters: stroke volume, left ventricular end-diastolic pressure (LVEDP), and right ventricular systolic pressure (RVSP). Stroke volume is calculated using the diameter of the left ventricular outflow tract (LVOT) and the velocity time integral (VTI) across it, with VTI often serving as a surrogate to minimize errors associated with LVOT diameter measurement. Early diastolic blood flow velocity across the mitral valve (E) and early diastolic mitral annular tissue velocity (e’) are also obtained, enabling estimation of LVEDP via the E/e’ ratio [33, 39]. RVSP is determined from the peak velocity of the tricuspid regurgitant jet. We propose a simplified, practical protocol that delivers a core set of hemodynamic parameters readily obtainable at the bedside to guide preload optimization in high-risk STEMI patients (Fig. 3).

When bedside echocardiography is performed in a high-risk post-STEMI patient to assess ventricular and valvular function, as well as to exclude mechanical complications and ventricular thrombus [32], quantitative Doppler assessment can be simultaneously included to document baseline LV and RV hemodynamics. In addition to physical assessment, laboratory results, and imaging studies, repeated Doppler evaluations and real-time hemodynamic monitoring in STEMI patients presenting with tachycardia and hypotension can uncover undetected right-left ventricular pressure discordance (R-L mismatch). These dynamic assessments may assist clinicians in identifying the causes of inadequate treatment response and inform critical management choices, including the use of inotropic support or fluid resuscitation. Echocardiography-based and hemodynamic-guided therapy have already be performed in practice by intensivists and anesthesiologists. In contrast to conventional clinical indicators like body weight and JVP assessmentt, real-time Doppler measurement can be more objective in assisting frontline physicians in judging instantaneous intravascular volume status, preventing over-diuresis that leads to hypotension, reflex tachycardia, and pre-renal dysfunction, so as to promptly commence ACEIs/ARNI, BBs, MRAs and SGLT2i in the early post-STEMI period. Timely Doppler-guided volume titration would also facilitate up-titration of the dosages of evidence-based pharmacotherapy before discharge, avoiding an extended medication inadequacy during the transition from inpatient to outpatient service. We suggested a stepwise approach integrating quantitative Doppler assessment into post-STEMI management workflow.

## Obstacles and prospects

Before quantitative Doppler can be routinely used to guide post-STEMI volume assessment and pharmacotherapy, potential obstacles should be explored, not the least of which is the lack of confidence or experience in performing and interpreting quantitative Doppler measurements in clinical services. Contrary to common belief, the skill set required for quantitative Doppler data acquisition is less prohibitive than a standard comprehensive echocardiography study. Technically interpretable data are usually obtained in a POCUS. It is well established that Doppler techniques can be quickly learned and accurately interpreted by clinicians across various specialties, including trainee physicians [40, 41]. Meanwhile, artificial intelligence (AI)-based image acquisition has already achieved essential advancements in both standardization and customization of ultrasonographic studies performed by bedside clinicians with minimal image training and experience [42]. Deep learning networks supporting software have been authorized by the U.S. Food and Drug Administration to provide real-time prescriptive guidance (turn-by-turn instructions) to novice operators for echocardiographic image acquisition [43]. The implementation of standardized protocols for acquiring, processing, and interpreting quantitative Doppler data will enhance its integration into everyday clinical practice and promote its adoption in future research studies [44–46].

Regarding concerns about the time required for quantitative Doppler measurements and the cost-effectiveness of repeated Doppler evaluations in monitoring hemodynamic changes during post-STEMI care, streamlined protocols and targeted application can enhance efficiency and clinical utility, making routine assessment more feasible in real-world practice. Since bedside echocardiography/POCUS is usually already needed in high-risk STEMI patients [32], simultaneous Doppler assessment would not be more time-consuming. Moreover, quantitative Doppler evaluations are employed selectively, only when standard clinical assessments and imaging fail to accurately determine real-time left ventricular preload status or clarify the reasons for inadequate treatment response. As a result, the Doppler-derived information is focused and purpose-driven. With predefined protocols and standardized acquisition sequences, these assessments can be conducted repeatedly in a time-efficient manner [41]. Conversely, the use of Doppler imaging may help avoid redundant diagnostic procedures and extended stays in the cardiac intensive care unit, thereby lowering the overall cost of post-STEMI management. But like all other examination methods, Doppler echocardiography has its limitations in application. For example, E/e’ is variable in patients with tachy- or bradycardia, atrial flutter/fibrillation. And it is not suitable for patients with severe mitral stenosis/regurgitation, annular calcification, surgical rings, prosthetic mitral valves, and constrictive pericarditis.

## Conclusions

Although novel intervention techniques, devices, and medications have substantially improved the prognosis of STEMI patients, the mortality rate still significantly varies among hospitals, and a substantial portion of high-risk STEMI patients continue to have a poor prognosis [1]. The concept of “time is muscle” applies for not only coronary revascularization, but also early pharmacotherapy. Although evidence-based pharmacotherapy in the post-STEMI period has been well proven to improve short- and long-term outcomes, real-world implementation of early pharmacotherapy remains suboptimal in high-risk patients who are miscategorized as being “intolerable” of GDMT. While POCUS has assumed increasingly greater roles in daily clinical practice, integrating real-time quantitative Doppler data can empower clinicians with instantaneous hemodynamic information to improve post-STEMI care quality, given established clinical algorithms and standardized imaging protocols. Further multi-center prospective studies would help define the roles of real-time quantitative Doppler in supporting early GDMT to achieve independent prognostic value in high-risk STEMI patients.

## Electronic Supplementary Material

Below is the link to the electronic supplementary material.

Supplementary Material 1

## Data Availability

No datasets were generated or analysed during the current study.
